# Supplementary material for: Basolateral amygdala neurons await oscillatory recruitment into valence-relevant ensembles
Source: Res Sq. 2026 Sep 18:rs.3.rs-10540538. Preprint. [Version 1] doi: 10.21203/rs.3.rs-10540538/v1 (PMC13596657; doi:10.21203/rs.3.rs-10540538/v1)
Supplement: Supplementary file 1 [file NIHPPrs10540538v1-supplement-1.pdf]

Supplemental Figures

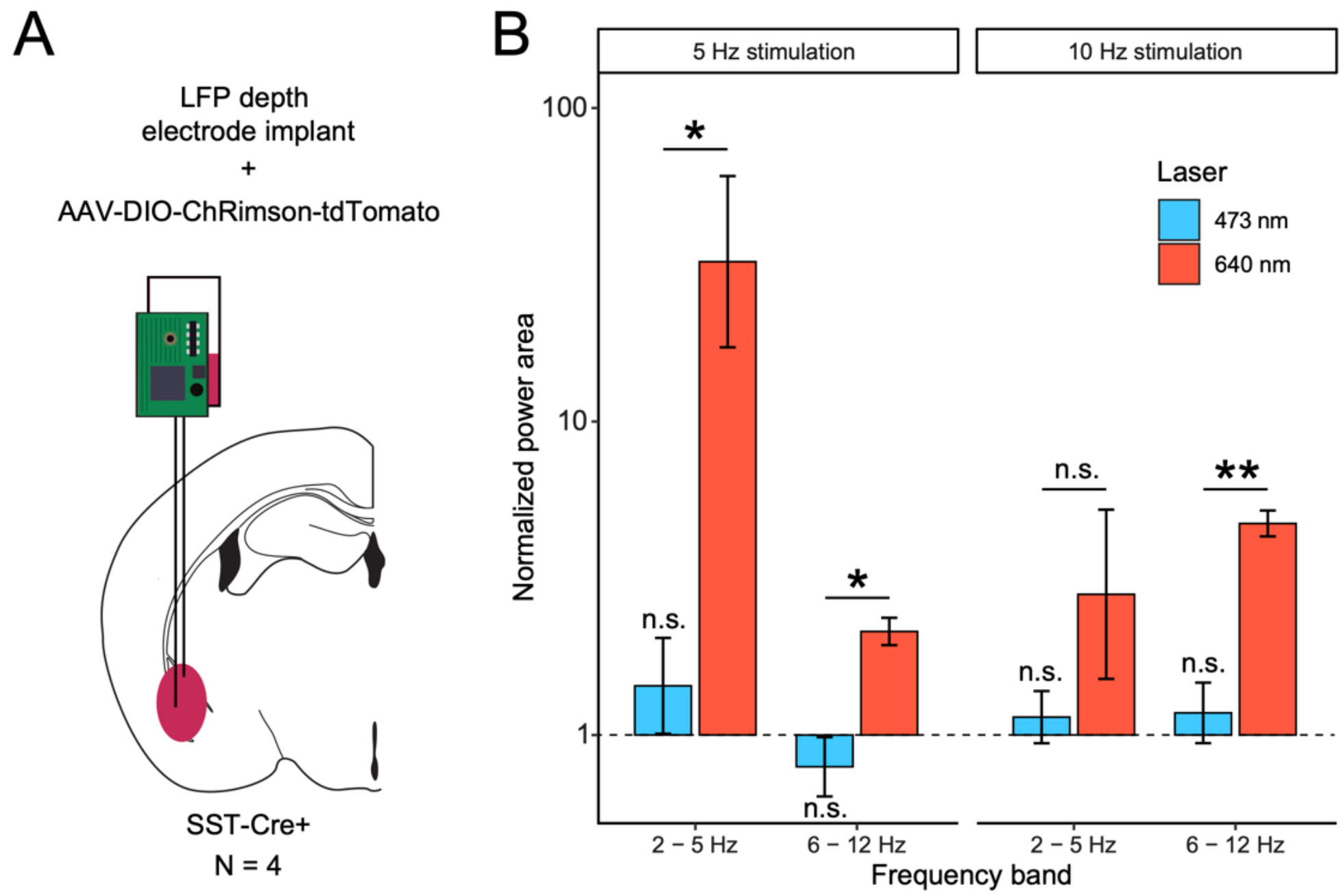

Supplemental Figure S1. Administration of blue light (473 nm) to ChRimson-expressing interneurons does not alter network states. A) Depiction of procedures where Cre-dependent ChRimson was infused into the BLA of SST-Cre+ mice (N = 4) followed by implantation of a depth electrode to record network activity. B) Normalized power area shown by stimulation frequency (5 Hz or 10 Hz stimulations), frequency band (low theta, 2 – 5 Hz; high-theta, 6 – 12 Hz), and laser wavelength (blue, 473 nm; red, 640 nm). Stimulating interneurons with the blue laser at 5 Hz had no effect on the normalized power area in low-theta, $t(3) = 1.03$, $p = 0.38$, and no effect on the normalized power in high-theta, $t(3) = -1.07$, $p = 0.36$. Similarly, stimulating interneurons with the blue laser at 10 Hz had no effect on the normalized power area in low-theta, $t(3) = 0.69$, $p = 0.54$, nor in high-theta, $t(3) = 0.73$, $p = 0.52$. We observed network changes when stimulating interneurons using the red laser. Specifically, when stimulating at 5 Hz, there is a significant difference between normalized power area between laser types in low-theta, $t(3.2) = -4.32$, $p = 0.02$, and in high-theta, $t(4.1) = -4.14$, $p = 0.013$. When stimulating interneurons at 10 Hz, we saw a significant difference between laser types in high theta, $t(4) = -5.76$, $p = 0.005$, but not in low-theta, $t(2.4)$. = -1.39, $p = 0.28$.

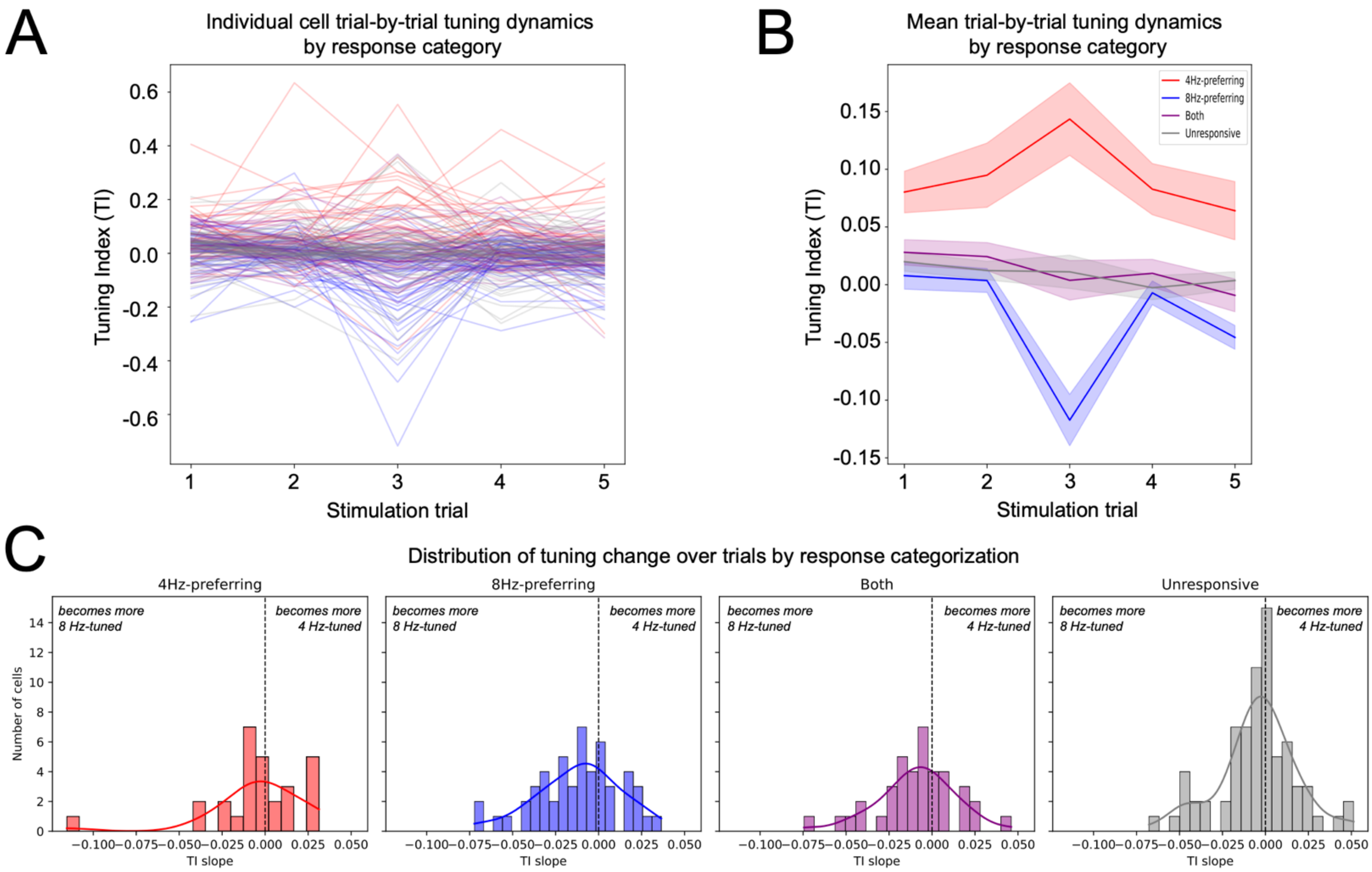

Supplemental Figure S2. Identified 4 Hz and 8 Hz preferring neurons do not increase or decrease their preference over stimulation trials. A) Tuning Index ([dF/F 4 Hz] – [dF/F 8 Hz]) shown over stimulation trials. Each line represents an individual cell, color coded by eventual preference categorization. B) Mean Tuning Index by response category with SEM shading. C) Histograms and density plots showing distributions of Tuning Index Slope separated by response categorization. Positive slopes represent an increase in 4 Hz tuning while negative slopes represent an increase in 8 Hz tuning. The distributions of identified cells are similarly centered across conditions.

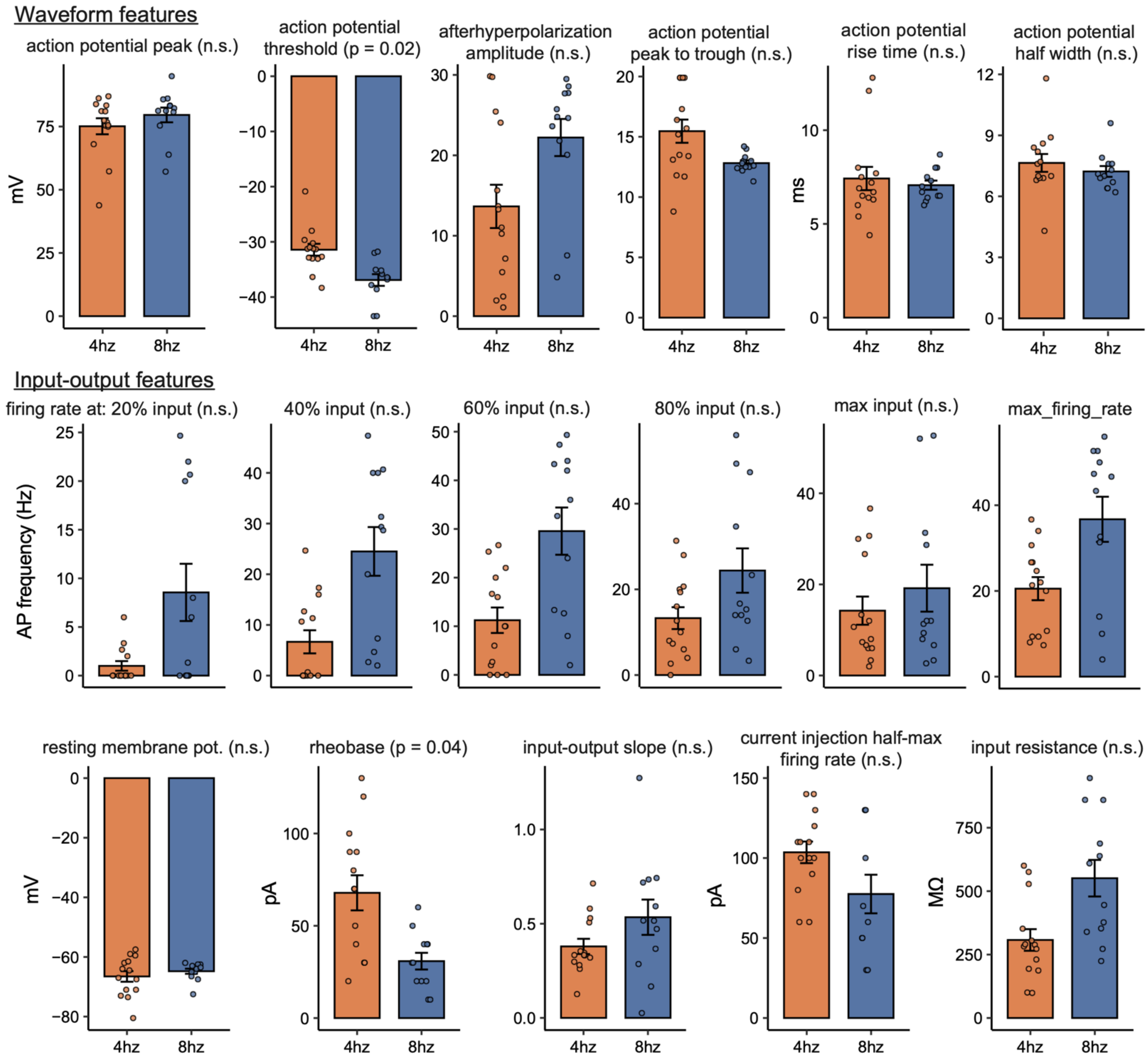

Supplemental Figure S3. Intrinsic electrophysiological properties of optogenetically-recruited neurons via interneuron stimulations at either 4 Hz (red) or 8 Hz (blue). Top: Waveform properties including, left to right, action potential peak, action potential threshold*, afterhyperpolarization amplitude, action potential peak to trough, action potential rise time, and action potential half-width. Bottom: Input-output features including firing rate at increasing input strengths: 20%, 40%, 60%, 80%, and maximum, as well as the maximum firing rate of recorded neurons. Bottom row, left to right: resting membrane potential, rheobase*, input-output slope, current injection half-max firing rate, and input resistance. * $p < 0.05$ after Bonferroni corrections for multiple comparisons.

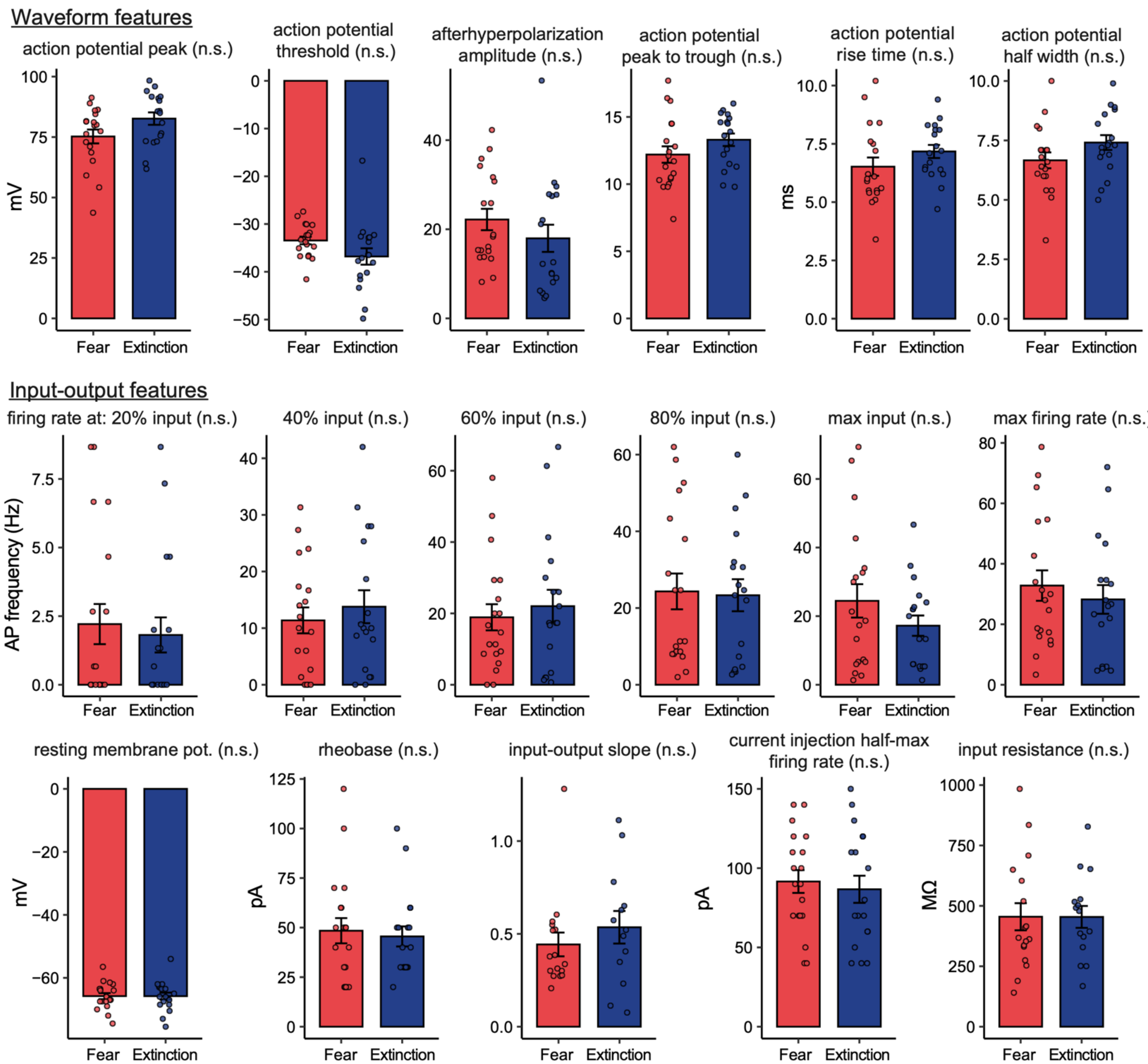

Supplemental Figure S4. Intrinsic electrophysiological properties of behaviorally-recruited neurons via fear conditioning (red) or extinction learning (blue). Waveform features: Top, Waveform properties including, left to right, action potential peak, action potential threshold, afterhyperpolarization amplitude. Bottom, action potential peak to trough, action potential rise time, and action potential half-width. Input-output features: Top, firing rate at increasing input strengths: 20%, 40%, 60%, 80%, and, Center, maximum, as well as the maximum firing rate of recorded neurons. Current injection half-max firing rate, input resistance, and resting membrane potential. Bottom, maximum firing rate, rheobase, and input-output slope. No significant effects observed.

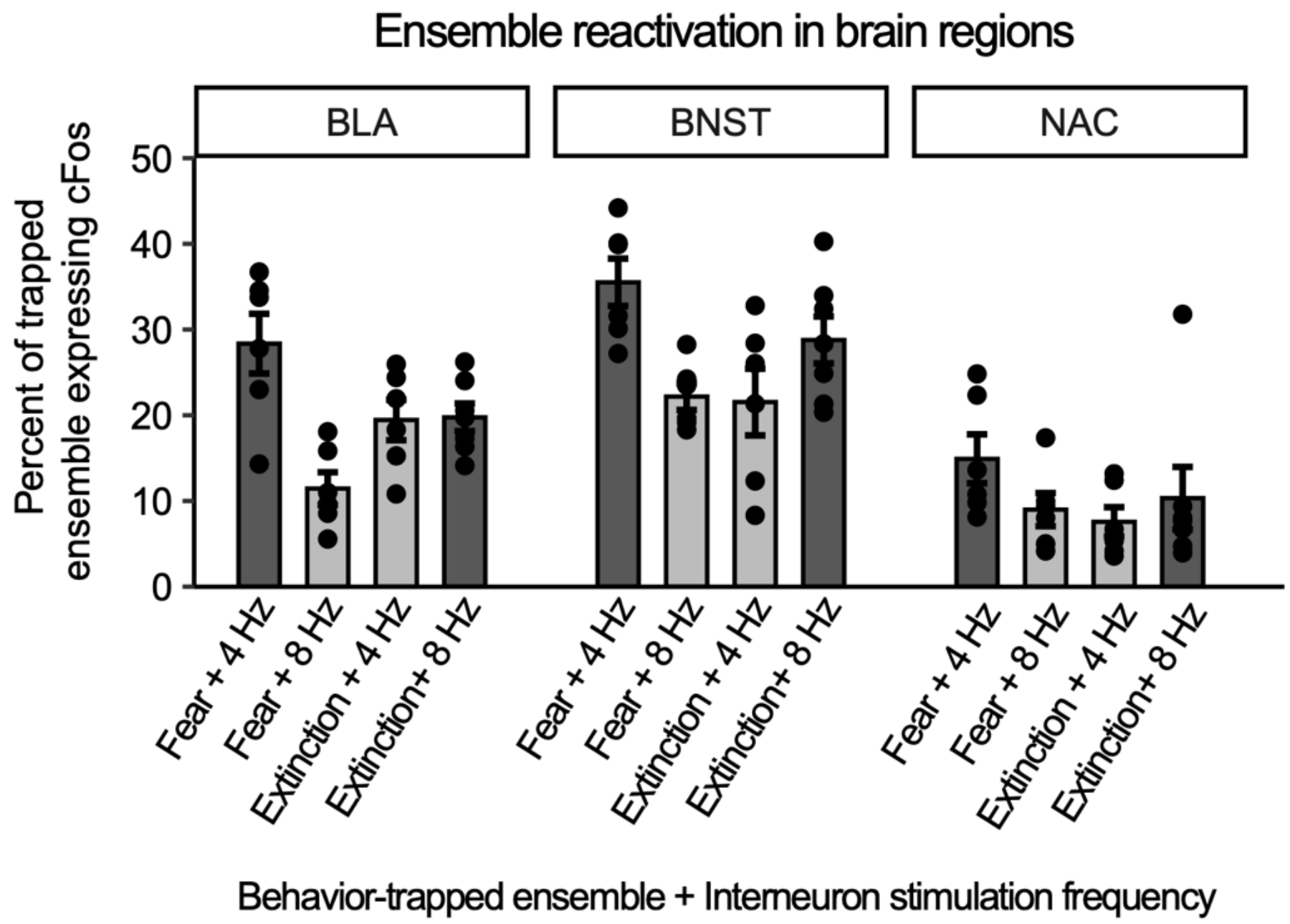

Supplemental Figure S5. Congruent conditions broken down into behavioral tag method (fear or extinction) and their combination with interneuron frequency stimulation (4 Hz or 8 Hz). Statistical analysis was conducted for data within each brain region, where generalized linear mixed models were used to model ensemble coexpression (coexpression/total_behavioral_ensemble) as a product of behavioral tag and stimulation frequency. For the BLA, there was no significant effect of stimulation frequency on coexpression (OR: 1.19; CI: 0.98 – 1.44; $p = 0.085$), but there was a significant effect of behavioral tag (OR: 11.04; CI: 1.97 – 61.82; $p = 0.006$). There was also a significant interaction between stimulation frequency and tag (OR: 0.74; CI: 0.57 – 0.96; $p = 0.024$). In the BNST, there were no significant effects observed among the behavioral tag (OR: 3.84; CI: 0.59 – 24.94; $p = 0.159$) nor stimulation frequency (OR: 1.13; CI: 0.92 – 1.39; $p = 0.244$), nor was there a significant interaction between these variables (OR: 0.88; CI: 0.66 – 1.19; $p = 0.412$). Similarly, in the NAc, there was not a significant effect of behavioral tag (OR: 6.94; CI: 0.28 – 172.56; $p = 0.238$) nor stimulation frequency (OR: 1.10; CI: 0.76 – 1.59; $p = 0.609$), nor was there a significant interaction between these variables (OR: 0.77; CI: 0.47 – 1.28; $p = 0.316$).

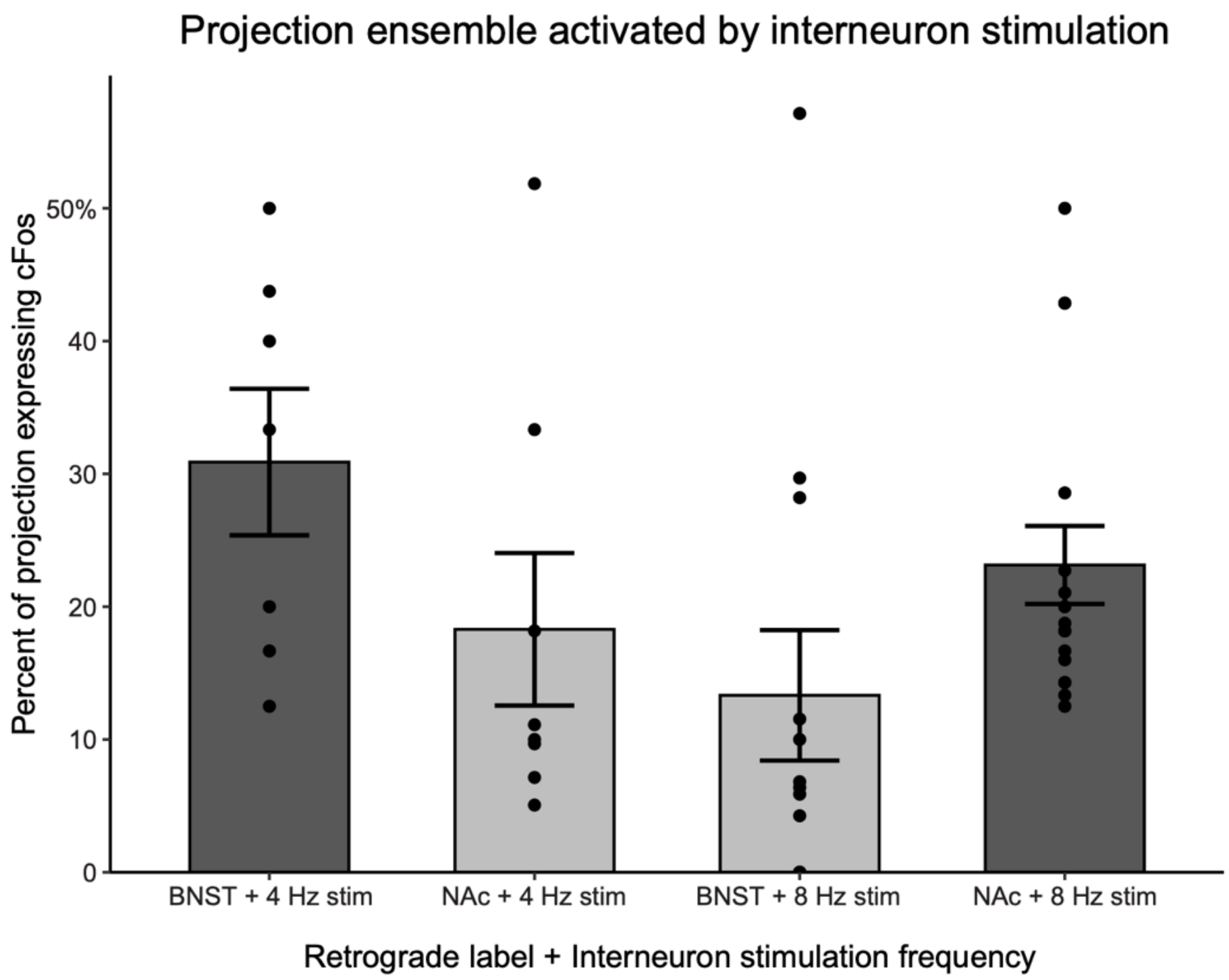

Supplemental Figure S6. Congruent conditions broken into retrograde label and interneuron stimulation pairings, colored by congruency. Statistical analysis was performed using a generalized linear mixed model weighted by total count of projection neurons observed in the BLA, (cFos coexpression/total) ~ label * stimulation + (1|animal_id), and family set to binomial. Results showed a significant effect of label on coexpression (OR: 0.11; CI: 0.01 – 0.88; p = 0.037) and a trending label by stimulation interaction (OR: 3.43; CI: 0.99 – 11.88; p = 0.052). There was no effect of interneuron stimulation frequency on coexpression (OR: 0.55; CI: 0.22 – 1.41; p = 0.214).

Supplemental Methods

Trial by trial calcium imaging response profiles

To quantify frequency selectivity on a trial-by-trial basis, a tuning index (TI) was computed for each neuron and trial as ($R_4Hz - R_8Hz$) / ($|R_4Hz| + |R_8Hz|$), where R_4Hz and R_8Hz represent stimulus-evoked responses. Trial-by-trial changes in tuning were assessed using ordinary least-squares linear regression of TI against trial number for each neuron independently, with the resulting slope parameter used as a measure of within-session response changes. Positive slopes indicate increasing 4 Hz selectivity over time, whereas negative slopes indicate a shift toward 8 Hz selectivity.

For visualization, neurons were sorted according to functional classification and mean stimulus-evoked response magnitude, and ΔF/F responses were organized into per-neuron, per-time-bin matrices separately for each stimulus frequency.

Intrinsic Properties Analyses

For input-output (IO) curve analysis, only cells that had more than 10 Hz maximum firing rate (5 APs in 0.5 s), were included. The IO slope was calculated using a first-degree least squares polynomial fit (NumPy's *polyfit*, *n* = 1) between rheobase to 90% of max firing rate. For the IO slope, cells were included only if they fired for at least 3 steps from rheobase to 90% of max APs (at least 3 points for line fit). For waveform analysis, only a maximum of 3 APs were collected from up to the first 3 current steps above rheobase. This was done, to ensure that the afterhyperpolarization (AHP) was maximally preserved. For all analyses, cells were excluded based on their maximum firing rate using Tukey's outlier detection method (k = 3). To calculate waveform properties, APs were interpolated (SciPy's *interp1d*, interpolation factor = 10, type = cubic). The AP threshold was detected as 1.5 mV/ms, the AP amplitude was defined as the difference from the AP peak – threshold. The AHP amplitude was defined as the absolute difference between the AHP peak – AP threshold. AP peak-to-trough was calculated as the time between AHP peak to AP peak. The rise time was calculated as the time between AP peak to AP threshold. Statistical tests were conducted in R with Bonferroni corrections for multiple comparisons. Figures were generated using python or R and stylized in Adobe Illustrator.
